# A Node Influence Based Label Propagation Algorithm for Community Detection in Networks

**DOI:** 10.1155/2014/627581

**Published:** 2014-06-04

**Authors:** Yan Xing, Fanrong Meng, Yong Zhou, Mu Zhu, Mengyu Shi, Guibin Sun

**Affiliations:** School of Computer Science and Technology, China University of Mining and Technology, Xuzhou, Jiangsu 221116, China

## Abstract

Label propagation algorithm (LPA) is an extremely fast community detection method and is widely used in large scale networks. In spite of the advantages of LPA, the issue of its poor stability has not yet been well addressed. We propose a novel node influence based label propagation algorithm for community detection (NIBLPA), which improves the performance of LPA by improving the node orders of label updating and the mechanism of label choosing when more than one label is contained by the maximum number of nodes. NIBLPA can get more stable results than LPA since it avoids the complete randomness of LPA. The experimental results on both synthetic and real networks demonstrate that NIBLPA maintains the efficiency of the traditional LPA algorithm, and, at the same time, it has a superior performance to some representative methods.

## 1. Introduction

In recent years, complex networks have been widely used in many fields, such as social networks, World Wide Web networks, scientist cooperation networks, literature networks, protein interaction networks, and communication networks [1, 2]. Extensive studies have shown that complex networks have the property of communities (modules or clusters), within which the interconnections are close, but between which the associations are sparse. This property reflects the extremely common and important topology structure of complex networks and it is very important for understanding the structure and function of complex networks.

A great number of community detection algorithms have been proposed in recent decades, including modularity optimization algorithms [3–5], spectral clustering algorithms [6–8], hierarchical partition algorithms [9, 10], label propagation algorithms (LPA) [11, 12], and information theory based algorithms [13]. Among them, LPA is by far one of the fastest community detection algorithms. The complexity of LPA algorithm is nearly linear time, and the design of the algorithm is simple, all of which make LPA algorithm receive quite a lot of attention from numerous scholars [14–17]. However, it still has a number of shortcomings; for example, the community detection results are unstable.

In this paper, we propose a novel node influence based label propagation algorithm for community detection in networks (NIBLPA), improving the performance of the traditional LPA algorithm by fixing the node sequence of label updating and changing the label choosing mechanism when more than one label is contained by the maximum number of nodes. Firstly, NIBLPA calculates the node influence value of each node as the importance measure of nodes on the networks and fixes the nodes updating sequence in the descending order of node influence value; secondly, NIBLPA processes the label propagation repeatedly until the community structure of networks is detected. During each label updating process, when more than one label returned with the maximum number of nodes, instead of randomly selecting one label, we introduce the label influence into label computing formula to reselect the label from the set of labels with the same maximum number of nodes to improve the stability. Finally, NIBLPA divides all nodes with the same label into a community. Extensive experimental studies, by using various networks, demonstrate that our algorithm NIBLPA can get better community detection results compared with the state-of-the-art methods.

The rest of this paper is organized as follows.  Section 2 introduces the related works including the traditional label propagation algorithm and the *k*-shell decomposition method. In Section 3, we introduce the main idea and the detailed process of our algorithm. The experimental results on various networks in Section 4 confirm the effectiveness of the algorithm. The conclusion is given in Section 5.

## 2. Related Work

A complex network can be modeled as a graph *G* = (*V*, *E*), where *V* = {*v*_1_, *v*_2_,…, *v*_*n*_} is the set of nodes, *E* = {*e*_1_, *e*_2_,…, *e*_*m*_} represents the edges between nodes, and *n* and *m* represent the number of nodes and edges in the network, respectively. Each edge in *E* has a pair of nodes in *V* corresponding. The label of *v*_*i*_ is denoted as *c*_*i*_. *N*(*i*) represents the neighborhood set of *v*_*i*_ and *d*_*i*_ is the degree of node *i*.

### 2.1. Label Propagation Algorithm for Community Detection in Networks

In 2007, Raghavan et al. [11] applied the label propagation algorithm (LPA) to community detection, and the main idea of LPA is to use the network structure as the guide to detect community structures. LPA starts by giving each node a unique label, such as integers and letters, and in every iteration, each node changes its label to the one carried by the largest number of its neighbors. If more than one label is contained by the same maximum number of its neighbors, then randomly select one from them. In this repeated process, the dense groups of nodes change their different labels into the same label and nodes with the same label will be grouped into the same community.

The following equation is the formula of label updating:

(1) $$\begin{array}{c} \underset{l}{c_{i}=\arg\;\max}\underset{j\in N^{l}(i)}{\sum }1, \end{array}$$

where *N* ^*l*^(*i*) represents the set of neighbors of *v*_*i*_ with label *l*.

For a weighted graph *G*, the weight of the edge between *v*_*i*_ and *v*_*j*_ is denoted as *w*_*ij*_ and the label updating formula is changed as follows:

(2) $$\begin{array}{c} \underset{l}{c_{i}=\arg\;\max}\underset{j\in N^{l}(i)}{\sum }w_{ij}. \end{array}$$

**Figure 1:**
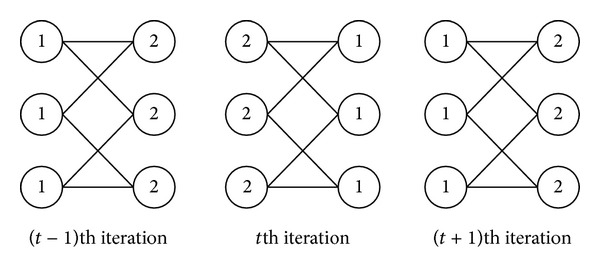
The oscillation of labels in a bipartite graph.

However, the algorithm cannot guarantee the convergence after several iterations. When the algorithm takes synchronize updating of the node labels (during the *t*th iteration, the node *x* adopts its label only based on the labels of its neighbors at the (*t* − 1)th iteration), oscillations will occur in bipartite or nearly bipartite graph. As shown in Figure 1, the labels on the nodes oscillate between *a* and *b* in a bipartite graph. Therefore, Raghavan et al. [11] proposed asynchronous updating where node *x* in the *t*th iteration updates its label based on a portion of labels at the *t*th iteration of its neighbors which have already been updated in the current iteration and another part of labels at the (*t* − 1)th iteration which are not yet updated in the current iteration to avoid the oscillation of labels.

The design of label propagation algorithm is simple and easy to be understood. The process of the algorithm is presented in Algorithm 1.

In large networks with a huge number of nodes, each time the network may have different divisions because of the randomness of LPA algorithm. Among the solutions, it is difficult to determine which is the optimal. So the stability issue of LPA is necessary to be settled.

### 2.2. The *k*-Shell Decomposition Method

There are many measures we usually use to calculate the node importance, such as degree centrality [21], clustering coefficient centrality [22], and betweenness centrality [23]. Degree and clustering coefficient of nodes can only characterize the local information of networks. The complexity of computing betweenness is very high due to the need to calculate the shortest path. Kitsak et al. [24] pointed out that nodes with large *k*-shell value are very important for spreading dynamics on networks.

A *k*-shell is a maximal connected subgraph of *G* in which every vertex's degree is at least *k*. The *k*-shell value of node *i*, denoted by *Ks*(*i*), indicates that node *i* belongs to a *k*-shell but not to any (*k* + 1)-shell. The *k*-shell decomposition method is often used to identify the core and periphery of networks. It starts by removing all nodes with only one link, until no such nodes remain and assigns them to the 1-shell. In the same manner, it recursively removes all nodes with degree 2 (or less), creating the 2-shell. The process continues, increasing *k* until all nodes in the network have been assigned to a shell. The shells with higher indices lie in the network core. The *k*-shell decomposition method can be efficiently implemented with the linear time complexity of *O*(*m*), where *m* is the number of edges in the network.

**Figure 2:**
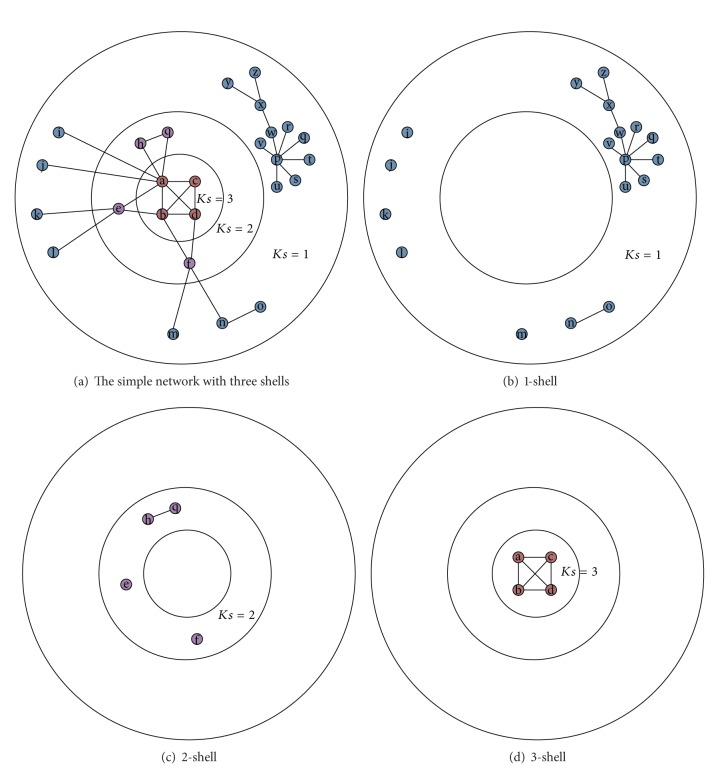
The illustration of *k*-shell decomposition method.

The *k*-shell decomposition method is shown in Figure 2. It is a simple network which can be divided into three different shells.

## 3. Our Method

Although asynchronous updating method can avoid oscillation of labels, there still are many limitations. As nodes are not updated simultaneously, the updating order of nodes plays a crucial impact on the stability and the quality of the results. The randomness of LPA in selecting one label when more than one label contained by the maximum number of nodes also makes the results unstable.

**Figure 3:**
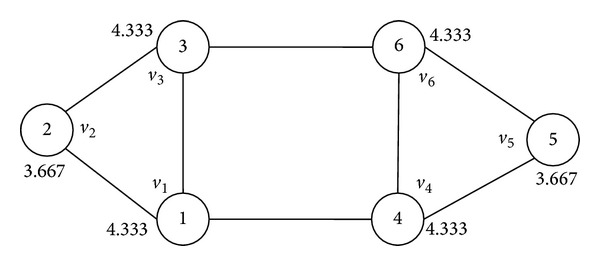
The toy sample network.

We analyze traditional LPA on a toy sample network in Figure 3 [25]. There are two communities in the network, {*v*_1_, *v*_2_, *v*_3_} and {*v*_4_, *v*_5_, *v*_6_}. The numbers inside the nodes represent their labels. Assuming that *v*_1_, *v*_2_, and *v*_3_ have already shared the same label 2, while *v*_4_, *v*_5_, and *v*_6_ still have unique labels. If we update *v*_4_ first and randomly choose label 2 as its new label, then update *v*_6_ before *v*_5_. As a consequence, all nodes are classified into the same community. On the other hand, if node *v*_4_ chooses label 6 and then updates node *v*_5_ before *v*_6_, the output will correspond with the right communities.

Seen from the above analysis, LPA is very sensitive to the node updating order and the label choosing method. In this section we propose solutions to overcome the issues discussed above to improve the traditional LPA algorithm.

### 3.1. The Basic Idea

In the new algorithm, we choose the asynchronous updating method to avoid oscillation of labels in Figure 1. But the randomly determined label updating order of nodes affects the stability of the algorithm. We should order the nodes based on their importance for the network and the more important nodes should be updated earlier.

A node with a big *k*-shell value indicates that it is located in the core of the network. However, in a network, there are too many nodes with the same *k*-shell value and we cannot rank the node effectively. In general, in a network a node with more connections to the neighbors located in the core of the network is more important for the network. Inspired by these previous studies, we propose a novel centrality measure by considering both the *k*-shell value and degree of node itself and its neighbor's *k*-shell values. The node influence of node *i* is defined as follows:

(3) $$\begin{array}{c} \text{NI}\left(i\right)=Ks\left(i\right)+\alpha *\underset{j\in N\left(i\right)}{\sum }\frac{Ks\left(j\right)}{d\left(j\right)}, \end{array}$$

where *α* is a tunable parameter from 0 to 1, which is used to adjust the effect of its neighbors on the centrality of node *i*.

We choose node influence value as the measure of node importance, so we arrange nodes in the descending order of node influence value. The fixed node updating sequence makes the algorithm more stable.

The other random factor causing the instability of LPA is that when the number of labels with maximum nodes is more than one, the algorithm randomly selects one of the labels to assign to the node. Instead of randomly selecting one of the labels contained by the maximum nodes, we improve the label updating formula using the information of the label influence.

The label influence of label *l* on node *i* is computed as follows:

(4) $$\begin{array}{c} \text{LI}\left(l\right)=\underset{j\in N^{l}\left(i\right)}{\sum }\frac{\text{NI}\left(j\right)}{d\left(j\right)}. \end{array}$$

The new formula of label updating is changed as follows:

(5) $$\begin{array}{c} c_{i}=\underset{l\in l\max}{\arg\;\max}\;\text{LI}\left(l\right), \end{array}$$

where *l*max⁡ denotes the set of labels that are simultaneously contained by the maximum nodes.

When multiple labels are simultaneously contained by the maximum nodes, we recalculate the value of the labels contained by the greatest number of nodes according to (5) and choose the label with the maximum value to assign to node *i*.

### 3.2. The Steps of NIBLPA Algorithm

The main steps of NIBLPA include initialization, iteration, and community division. Then NIBLPA can be described as Algorithm 2.

**Figure 4:**
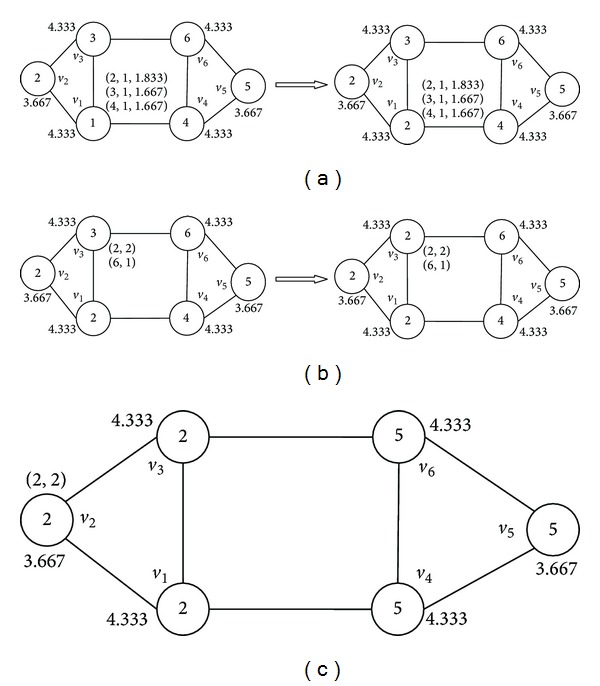
Label propagation process of NIBLPA.

We implement NIBLPA on the toy sample network in Figure 3 with *α* = 1. The decimals outside the nodes are the node influence value. Using our method on this network, the node updating sequence is fixed as *v*_1_-*v*_3_-*v*_4_-*v*_6_-*v*_2_-*v*_5_ (when NI(*i*) = NI(*j*), rank *i* and *j* by their node IDs). The label propagation process is shown in Figure 4.

Firstly, we update the label of node *v*_1_. We label *v*_1_ with a set of tuples (*l*, *n*, LI(*l*)), where *c* is a label contained by its neighbor, and *n* represents the number of its neighbors having the label *l*, and LI(*l*) is an optional value recalculated by (5) when multiple labels are contained by the maximum neighbors. As shown in Figure 4(a), *v*_1_ has three neighbors and they all have different labels with each other, and the set of tuples is {(2,1, 1.833), (3,1, 1.667), (4,1, 1.667)}. So we choose label 2 as its new label.

Then, node *v*_3_ is the next. After the label updating of *v*_1_, there are two neighbors of *v*_3_  that share label 2 and only one contains label 6, so we relabel *v*_3_ with label 2 as shown in Figure 4(b). The next label propagations of *v*_4_ and *v*_6_ are consistent with *v*_1_ and *v*_3_.

Now only *v*_2_ and *v*_5_ are not updated and, as shown in Figure 4(c), all of their neighbors contain the same labels with themselves, respectively, so we do not need to relabel them. After only one iteration using this method, we get the final solution that contains two communities exactly the same with the ground truth. Since there is no randomness, the outcome is deterministic and perfect.

### 3.3. Time Complexity

The time complexity of the algorithm is estimated below. *n* is the number of nodes, and *m* is the number of edges.

1. The time complexity of initialization for all nodes: *O*(*n*).
2. The time complexity of calculating the node influence value of all nodes: *O*(*m*).
3. The time complexity of ranking the nodes in descending order of NI: *O*(*n* log (*n*)).
4. Each iteration of label propagation consists of two parts:
  1. the time complexity of normal label updating: *O*(*m*);
  2. the time complexity of recalculating the labels based on (5) if necessary: *O*(*m*).
5. The time complexity of assigning the nodes with the same label to a community: *O*(*n*).

Phases (3) are repeated, so the time complexity of the whole algorithm is 2 × *O*(*n*)+(2 × *t* + 1) × *O*(*m*) + *O*(*n*log⁡ (*n*)), where *t* is the number of iterations and it is a small integer.

## 4. Experimental Studies

This section evaluates the effectiveness and the efficiency of our algorithm. We compare the performance of NIBLPA with LPA, KBLPA, and CNM. Where KBLPA is an improved LPA algorithm changing the node updating sequence by descending order of *k*-shell value. All the simulations are carried out in a desktop PC with Pentium Core2 Duo 2.8 GHz processor and 3.25 GB memory under Windows 7 OS. We implement our algorithm in Microsoft Visual Studio 2008 environment.

### 4.1. Datasets

In this section, we choose two types of synthetic and eight real networks to make experiments.

#### 4.1.1. Clique-Ring Networks

**Figure 5:**
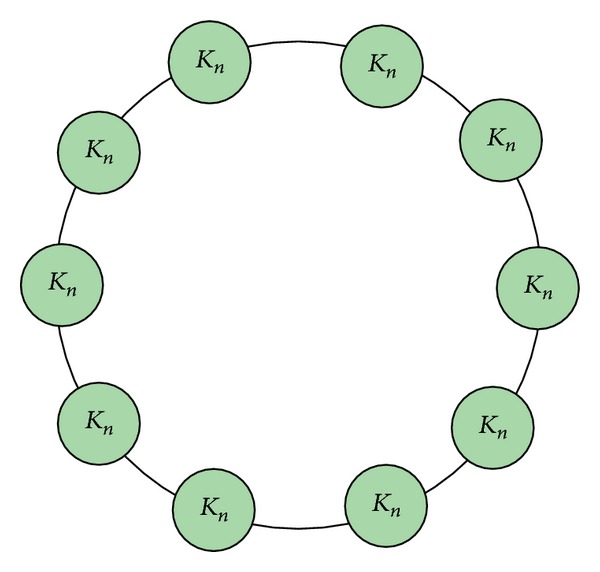
The structure of Clique-Ring networks.

Clique-Ring networks [26]: each clique is a complete subgraph *K*_*n*_ consisting of *n*  (*n* ≥ 3) nodes and *n*(*n* − 1)/2 edges. A Clique-Ring network composed of *m* such as subgraphs (*m* ≥ 2) has *N* = *mn* nodes and *M* = *mn*(*n* − 1)/2 + *m* edges. The structure is shown in Figure 5.

According to the generation rules of Clique-Ring networks, we construct four different size Clique-Ring networks. The parameters are shown in Table 1.

#### 4.1.2. LFR Benchmark Networks

LFR benchmark networks [27, 28] are currently the most commonly used synthetic networks in community detection. It can generate networks based on users' need by changing the following parameters in Table 2.

We generate six groups of LFR benchmark networks and all the networks share the common parameters of max⁡*k* = 50. Each group contains nine networks with mu ranging from 0.1 to 0.9 and they also share parameters *N*, *k*, min⁡*c*, and max⁡*c*, respectively. The other parameters are set to the default values. The details are shown in Table 3.

#### 4.1.3. Real Networks

We also make experiments on eight well known real networks, including Zachary's karate club networks, Dolphins social networks, and American College Football networks. The detailed information of each network is shown in Table 4.

### 4.2. Evaluation Criteria

In this paper, we use modularity (*Q*) [2], *F*-measure [29], and normalized mutual information (NMI) [30] as the evaluation criteria which are currently widely used in measuring the performance of network clustering algorithms. Computing *F*-measure and NMI needs to know the true community structure of the network, while the modularity does not. For synthetic networks, since the ground truth of the community structure has been known, we use both *F*-measure and NMI on Clique-Ring networks and LFR benchmark networks to evaluate the results of community detection. While the underlying class labels of most real networks are unknown, we can only adopt the modularity as the evaluation criteria on partial real networks and use both NMI and modularity on others with known community structure.

#### 4.2.1. Modularity

Consider the following:

(6) $$\begin{array}{c} Q=\frac{1}{2m}\overset{}{\underset{i,j\in V}{\sum }}(A_{ij}-\frac{d_{i}d_{j}}{2m})\times \delta \left(c_{i},c_{j}\right), \end{array}$$

where *m* represents the number of edges in the network; *A* is the adjacency matrix of the network, if node *i* and node *j* are directly connected, *A*_*ij*_ = 1; otherwise, *A*_*ij*_ = 0; *c*_*i*_ and *c*_*j*_, respectively, denote the label of node *i* and node *j*, if *c*_*i*_ = *c*_*j*_, then *δ*(*c*_*i*_, *c*_*j*_) = 1, else *δ*(*c*_*i*_, *c*_*j*_) = 0.

#### 4.2.2. *F*-Measure

Consider the following:

(7) $$\begin{array}{c} F\text{-Measure}=\frac{2\times \text{Precision}\times \text{Recall}}{\text{Precision}+\text{Recall}}, \end{array}$$

where precision and recall are written as (8), respectively,

(8) $$\begin{array}{c} \text{Precision}=\frac{\left|S\cap T\right|}{\left|S\right|},\\ \text{Recall}=\frac{\left|S\cap T\right|}{\left|T\right|}. \end{array}$$

*T* is the set of node pairs (*i*, *j*), where nodes *i* and *j* belong to the same classes in the ground truth, and *S* is the set of node pairs that belong to the same clusters generated by the evaluated algorithm. Then *S*∩*T* represents the intersection of node pairs of the ground truth and the clustering result.

#### 4.2.3. Normalized Mutual Information (NMI)

Consider the following:

(9) NMI(X ∣ Y) =(−2×∑i=1|X| ∑j=1|Y||Xi∩Yj|×log⁡⁡((n×|Xi∩Yj|)(|Xi|×|Yj|)))  ×(∑i=1|X||Xi|log⁡⁡(|Ci|n)+∑j=1|Y||Yi|log⁡⁡(|Cj|n))−1,

where *n* represents the number of nodes in the network, *X* represents a community detection result generated by the evaluated algorithm, and *Y* represents the ground truth community structure.

### 4.3. Experimental Results and Analysis

In this section, the synthetic and real networks are used to test the effectiveness of NIBLPA comparing with traditional LPA, KBLPA, and CNM. Where LPA and KBLPA are processed 100 times and the average value is used as the results because of the randomness of these algorithms. We compare the stability of the algorithms by analyzing the fluctuation range of all the results.

#### 4.3.1. The Experiments on Clique-Ring Networks

Table 5 shows the comparative results of the four algorithms on four different Clique-Ring networks, and for each instance, the best results are presented in boldface. The *F*-measure and NMI of LPA and KBLPA are in the form of average value ± the maximum difference between one result and the average value.

It can be seen from Table 5 that in the Clique-Ring networks which have special structure, NIBLPA can exactly detect the correct communities and CNM gets the right community structure on the first three networks. But on network C4, the result of CNM is much worse than others because modularity has the resolution limit problem. While the average *F*-measure of KBLPA algorithm is the lowest among LPA, KBLPA, and NIBLPA on the four networks and the average NMI of KBLPA is the lowest on most of the four networks except C4. These results illustrate that the fixed node sequence descending by the *k*-shell value at each step of label propagation cannot get good results. The instability of KBLPA is caused by the randomness of selecting label when multiple labels are simultaneously contained by the greatest number of nodes.

#### 4.3.2. The Experiments on LFR Benchmark Networks

**Figure 6:**
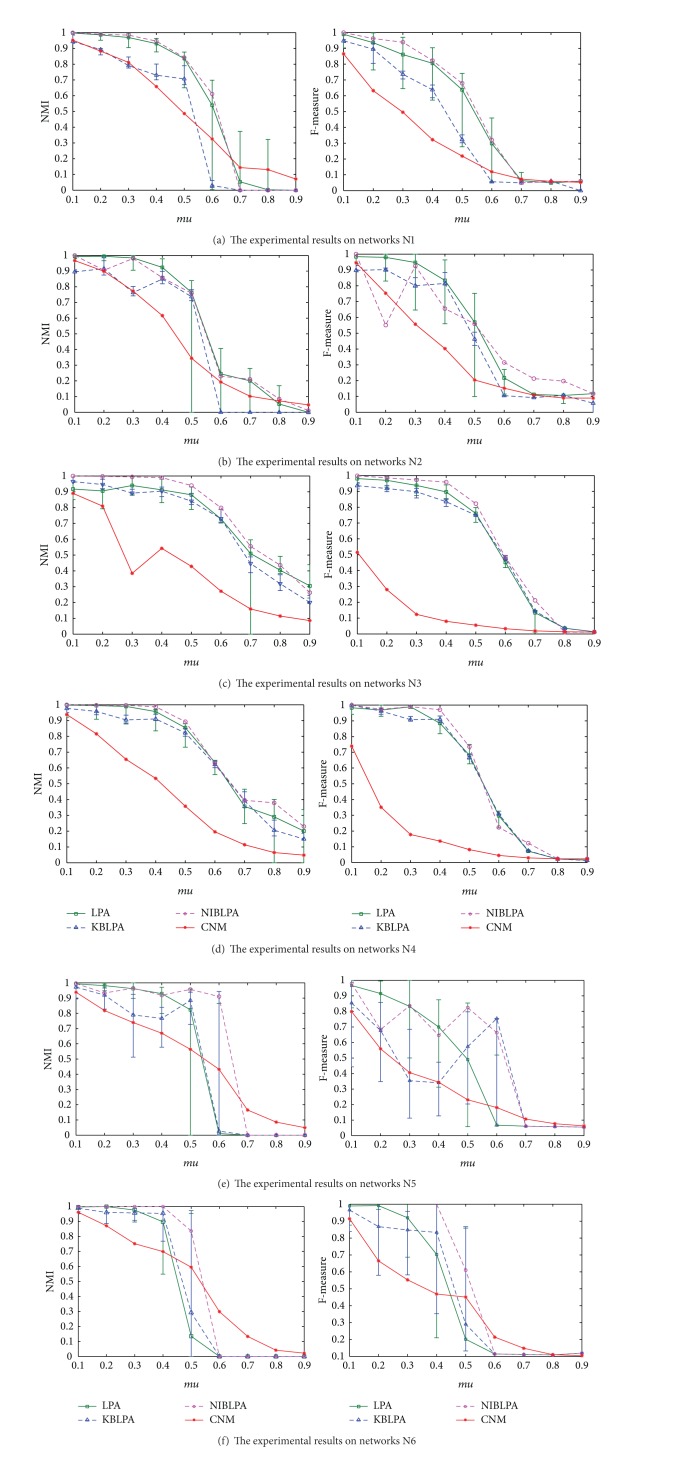
The results of the four algorithms on LFR benchmark networks.

The twelve figures in Figure 6 are the NMI and *F*-measure of the four algorithms on six groups of LFR benchmark networks (N1~N6). The abscissa represents the parameter *mu* from 0.1 to 0.9. The ordinate in the left figures is the NMI of the results and the ordinate in the right figures is the *F*-measure.

The twelve figures in Figure 6 show that with the increase of *mu*, the network structure is more and more complex and the four algorithms cannot be effective to detect the community structure. When mu is especially larger than 0.5, the NMI and *F*-measure decrease quickly. But generally, the performance of NIBLPA is better than the other three algorithms. Although NIBLPA does not guarantee to get the best performance, it can return stable, unique, and satisfied results. It can also be seen in Figure 6 that the fluctuation range of NMI and *F*-measure of LPA algorithm is large. KBLPA is also relatively stable, but its results are worse than LPA and NIBLPA. On these complex networks, CNM algorithm cannot detect the network structure effectively and it generally gets less number of communities than the truth.

#### 4.3.3. The Experiments on Different Sizes of Networks

In order to compare the time efficiency of the algorithms, we generate 10 LFR benchmark networks, the size of which is from 1,000 to 10,000, and the other parameters are the same (*k* = 10, max⁡*k* = 50, min⁡*c* = 10, max⁡*c* = 50, and *mu* = 0.1).

**Figure 7:**
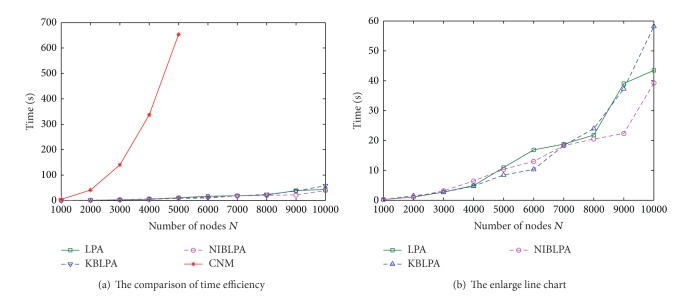
The efficiency comparison of the four algorithms on different sizes of networks.

The time consumption of the four algorithms on the 10 LFR benchmark networks is shown in Figure 7(a).  Figure 7(b) is the enlarge line chart of LPA, KBLPA, and NIBLPA.

From Figure 7, it is observed that the four algorithms use more and more time with the increase of the size of networks and CNM uses the longest time. When the number of nodes *N* is larger than 5000, CNM cannot get the community structure because of the limit of computer memory. From Figure 7(b), one can note that when the number of nodes is greater than 7000, the time consumption of NIBLPA is less than LPA. To some extent, we can say NIBLPA is more suitable for community detection on large scale networks.

#### 4.3.4. The Experiments on Real Networks

The eight real-world networks shown in Table 4 are commonly employed in the community detection literature and the first four networks have known ground truth community structures. So we compare the modularity *Q* and normalized mutual information NMI on the first four networks and only compare the modularity *Q* on the last four networks.

Table 6 shows the experimental results on the eight real networks, and for each instance, the best *Q* and NMI are presented in boldface.

It can be seen from Table 6 that in all the real networks besides R7(Blog) and R8(PGP), the modularity of NIBLPA is higher than the other three algorithms. Simultaneously, the NMI of NIBLPA on the first four networks is the best. The stability of KBLPA is better than LPA, but the modularity and NMI of KBLPA are worse than LPA on almost all of the networks. On the large size of PGP-network, CNM cannot detect the community structure. In general, NIBLPA can get better and stable results than the other three algorithms.

#### 4.3.5. Instance Analysis

We compare the community structure detected by NIBLPA when NMI achieves the maximum with the true community structure of Dolphins.

**Figure 8:**
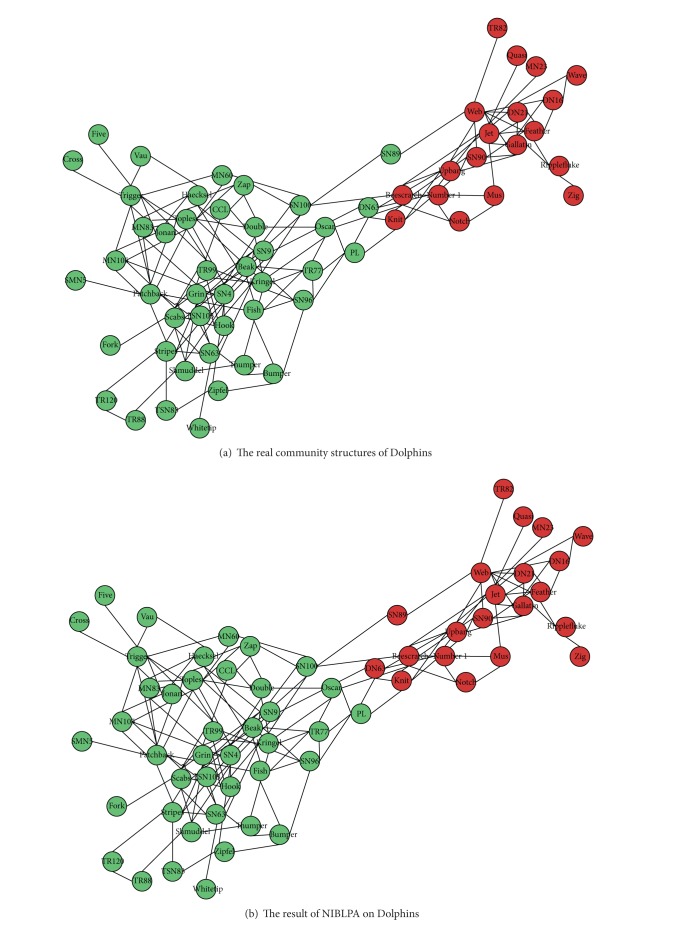
The community structures of Dolphins.

Figure 8(a) shows the real community structures of Dolphins and Figure 8(b) is a community detection result of NIBLPA on Dolphins. Comparing these two figures, the division of DN63 and SN90 based on NIBLPA is inconsistent with the real structure. From the topology structure of Dolphins, we can see that DN63 has two adjacent nodes and they, respectively, belong to the two communities; DN63 has five neighbors, NIBLPA algorithm assigns it to the community which its most neighbors belong to. The modularity of Dolphins real community structure is lower than the result of NIBLPA, which draws a conclusion that the community division of NIBLPA is a reasonable result.

#### 4.3.6. Parameter Selection

There is only one parameter in NIBLPA algorithm, tunable parameter *α*. In order to analyze the impact of the parameter, we run NIBLPA with different values of *α* on synthetic networks and compare NMI to analyze the effect of the parameter on the algorithm. In this way, we can investigate that under which *α* the NIBLPA can achieve the best results.

**Figure 9:**
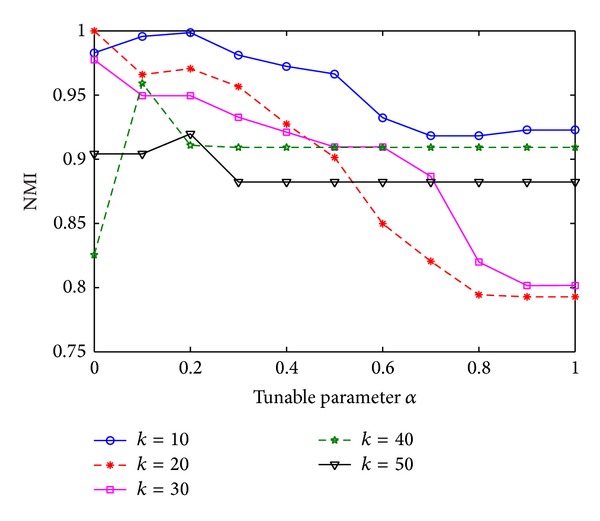
The value of NMI under different tunable parameter *α*.

We generate five LFR benchmark networks with *k* ranging from 10 to 50 and all the networks share the common parameters of *N* = 1000, max⁡*k* = 50, min⁡*c* = 10, max⁡*c* = 50, and *mu* = 0.1.  Figure 9 shows the results of NIBLPA on these networks.

As it can be seen in Figure 9, under different parameter *α*, the value of NMI changed a lot. However, for each network, there is an optimal *α* under which the NIBLPA method can achieve the largest NMI. Moreover, on each network, the first extreme large value is generally the best result.

## 5. Conclusion

This paper presents a node influence based label propagation algorithm for community detection in networks. The algorithm firstly calculates the node influence value for each node and ranks the node in the descending order of node influence value. During each label updating process, when more than one label is contained by the maximum number of nodes, we introduce the label influence value into the formula of label updating to improve the stability. After the algorithm converges, nodes with the same label are divided into a community. This algorithm maintains the advantages of the original LPA algorithm. Moreover, it can get the stable community detection results by avoiding the randomness of label propagation. By experimental studies on synthetic and real networks, we demonstrate that the proposed algorithm has better performance than some of the current representative algorithms.

## Figures and Tables

**Algorithm 1:**
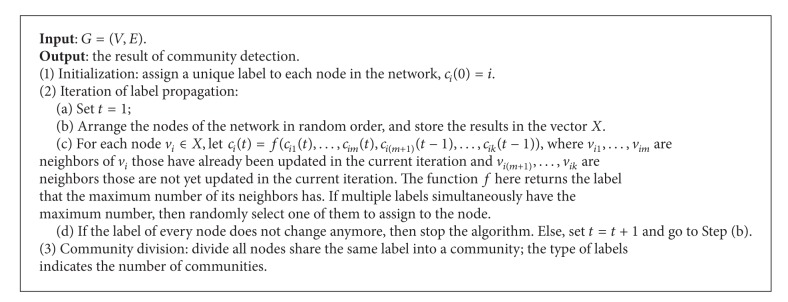
Label propagation algorithm for community detection in networks (LPA).

**Algorithm 2:**
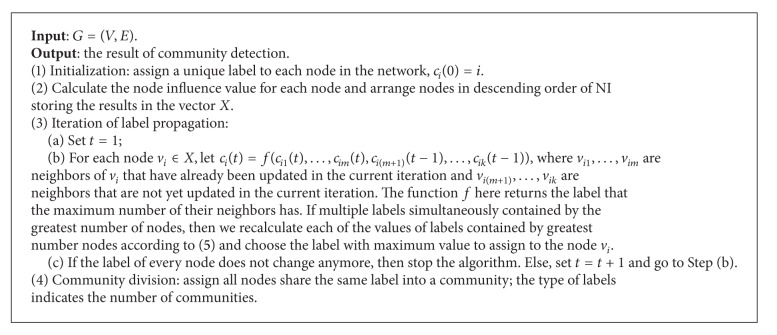
Node influence based label propagation algorithm for community detection in networks (NIBLPA).

**Table 1:** The parameters of four Clique-Ring networks.

Network ID	Dataset	*n*	*m*	Number of nodes	Number of edges	Number of communities
C1	Clique-Ring1	5	5	25	55	5
C2	Clique-Ring2	5	10	50	110	10
C3	Clique-Ring3	10	10	100	460	10
C4	Clique-Ring4	5	30	150	330	30

**Table 2:** The main parameters of LFR.

Parameters	Meaning
*N*	The number of nodes
*k*	The average degree
max⁡⁡*k*	The maximum degree
*γ*	The exponent for the degree distribution
*β*	The exponent for community size distribution
*mu*	The mixing parameter for the topology
min⁡⁡*c*	The minimum for the community sizes
max⁡⁡*c*	The maximum for the community sizes

**Table 3:** The parameters of six groups of LFR networks.

Networks	*N*	*k*	max⁡⁡*k*	min⁡⁡*c*	max⁡⁡*c*	*mu*
N1	1000	10	50	10	50	0.1~0.9
N2	1000	10	50	20	100	0.1~0.9
N3	5000	10	50	10	50	0.1~0.9
N4	5000	10	50	20	100	0.1~0.9
N5	1000	20	50	10	50	0.1~0.9
N6	1000	20	50	20	100	0.1~0.9

**Table 4:** The information of real networks.

Network ID	Network name	Number of nodes	Number of edges	Number of communities	References
R1	Karate	34	78	2	[18]
R2	Dolphins	62	159	2	[18]
R3	Political Books	105	441	3	[18]
R4	Football	115	613	12	[18]
R5	Email	1133	5451	—	[19]
R6	Netscience	1589	2742	—	[20]
R7	Blogs	3982	6803	—	[19]
R8	PGP	10680	24316	—	[19]

**Table 5:** The comparison of results on Clique-Ring networks.

Networks	*F*-measure				NMI			
	LPA	KBLPA	NIBLPA	CNM	LPA	KBLPA	NIBLPA	CNM
C1	0.959 ± 0.39	0.937 ± 0.45	**1**	**1**	0.988 ± 0.24	0.954 ± 0.05	**1**	**1**
C2	0.971 ± 0.15	0.949 ± 0.38	**1**	**1**	0.992 ± 0.07	0.987 ± 0.12	**1**	**1**
C3	0.995 ± 0.10	0.988 ± 0.13	**1**	**1**	0.999 ± 0.03	0.996 ± 0.07	**1**	**1**
C4	0.965 ± 0.17	0.960 ± 0.29	**1**	0.615	0.995 ± 0.03	0.997 ± 0.02	**1**	0.887

**Table 6:** The comparison of results on real networks.

Network ID	*Q*				NMI			
	LPA	KBLPA	NIBLPA	CNM	LPA	KBLPA	NIBLPA	CNM
R1	0.296 ± 0.29	0.073 ± 0.23	**0.423**	0.345	0.583 ± 0.58	0.128 ± 0.88	**1**	0.479
R2	0.465 ± 0.19	0.489 ± 0.12	**0.521**	0.306	0.516 ± 0.18	0.471 ± 0.22	**0.622**	0.423
R3	0.489 ± 0.15	0.449 ± 0.09	**0.497**	0.265	0.572 ± 0.07	0.528 ± 0.07	**0.656**	0.231
R4	0.582 ± 0.14	0.573 ± 0.09	**0.582**	0.537	0.863 ± 0.24	0.849 ± 0.13	**0.872**	0.698
R5	0.380 ± 0.27	0.183 ± 0.34	**0.427**	0.415	—	—	—	—
R6	0.871 ± 0.03	0.883 ± 0.02	**0.899**	0.631	—	—	—	—
R7	0.791 ± 0.02	0.808 ± 0.01	0.775	**0.849**	—	—	—	—
R8	0.806 ± 0.02	0.775 ± 0.03	0.783	—	—	—	—	—
